# The Football Sign: An Alarming Feature on Supine Radiograph

**DOI:** 10.7759/cureus.12867

**Published:** 2021-01-22

**Authors:** Hsin-Ti Lin, Chiao-Jen Cheng, Teressa Ju, Alexander L Wang, Wei-cheng Chen

**Affiliations:** 1 Department of Internal Medicine, China Medical University Hospital, Taichung, TWN; 2 Department of Internal Medicine, NewYork-Presbyterian Queens, New York, USA; 3 Division of Pulmonary and Critical Care Medicine, China Medical University Hospital, Taichung, TWN

**Keywords:** football sign, pneumoperitoneum, hollow organ perforation

## Abstract

Football sign is a rare radiographic finding on abdominal x-ray that suggests massive pneumoperitoneum. Free air outlines the abdominal cavity and falciform ligament which creates the radiolucent oval contour of a football. Football sign is hardly reported in older children or adults. We present the first clear image of football sign caused by gastric perforation in an adult patient. A 57-year-old male with a history of hepatocellular carcinoma was diagnosed with an undrainable liver abscess and partial gastric outlet obstruction. He developed acute onset of severe abdominal pain afterward and abdominal plain film showed a large oval radiolucency over the central part of the abdomen without interruption by intestine, a classical finding of pneumoperitoneum also known as a “football sign”. Emergent laparotomy revealed a 0.5 cm perforation hole at the anterior surface of the gastric antrum. Despite timely intervention, the patient died from progression of multiorgan failure. This case describes an alarming radiographic finding that rarely occurs in the adult population. Air could be identified on x-ray in this patient due to presence of massive ascites in his abdominal cavity. Recognizing radiographic patterns that suggest pneumoperitoneum on supine plain radiographs could expedite the diagnostic process and surgical intervention.

## Introduction

Non-traumatic gastrointestinal (GI) preformation is a life-threatening condition that requires timely diagnosis and intervention [1]. Presence of extraluminal air, as well as certain radiographic features, on plain radiographs may be the initial clue to the diagnosis [2]. Football sign is an infrequent radiologic finding of massive pneumoperitoneum seen on supine plain radiographs. In the supine position, free air gathers below the abdominal wall and forms a large bubble that distends the peritoneal cavity and creates the radiolucent oval contour of a football [3]. Football sign most commonly occurs in neonates [4-8] and is rarely reported in older children or adults. We present a 57-year-old man who had a clear image of football sign due to gastric perforation.

## Case presentation

A 57-year-old man with a history of hepatocellular carcinoma first presented to the emergency department with fever for 10 days. Abdominal computed tomography (CT) revealed a distended stomach, a subphrenic liver tumor, and a fluid collection with free air at the perihilar area of the liver, indicative of tumor necrosis with abscess formation. Esophagogastroduodenoscopy demonstrated gastric ulcers and a protruding mass at the antrum of the stomach causing gastric outlet obstruction. A diagnosis of liver tumor necrosis with abscess formation and partial gastric outlet obstruction was made. Since the abscess was located adjacent to the hilar vessels, drainage of the abscess was not attempted. Fever and abdominal pain improved after five days of flomoxef, and he was discharged with oral antibiotics.

**Figure 1:**
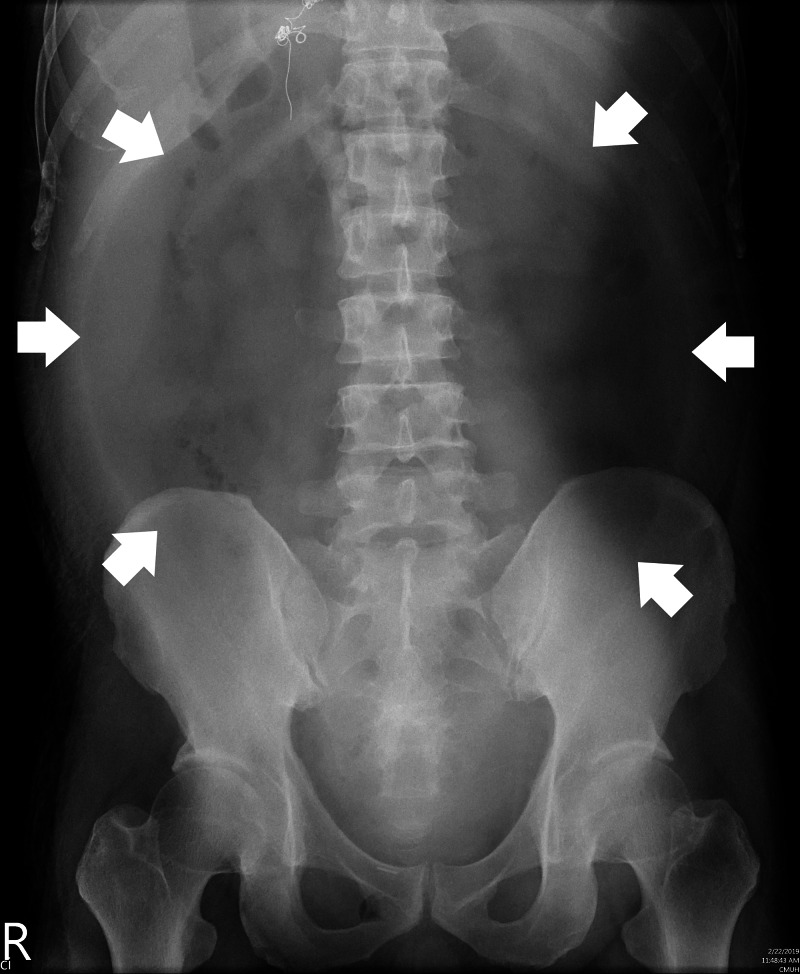
Football sign Football sign in a patient with acute abdominal pain. A vertically oriented oval radiolucency in the central part of the abdomen (white arrows) indicating pneumoperitoneum can be seen on this supine abdominal x-ray.

**Figure 2:**
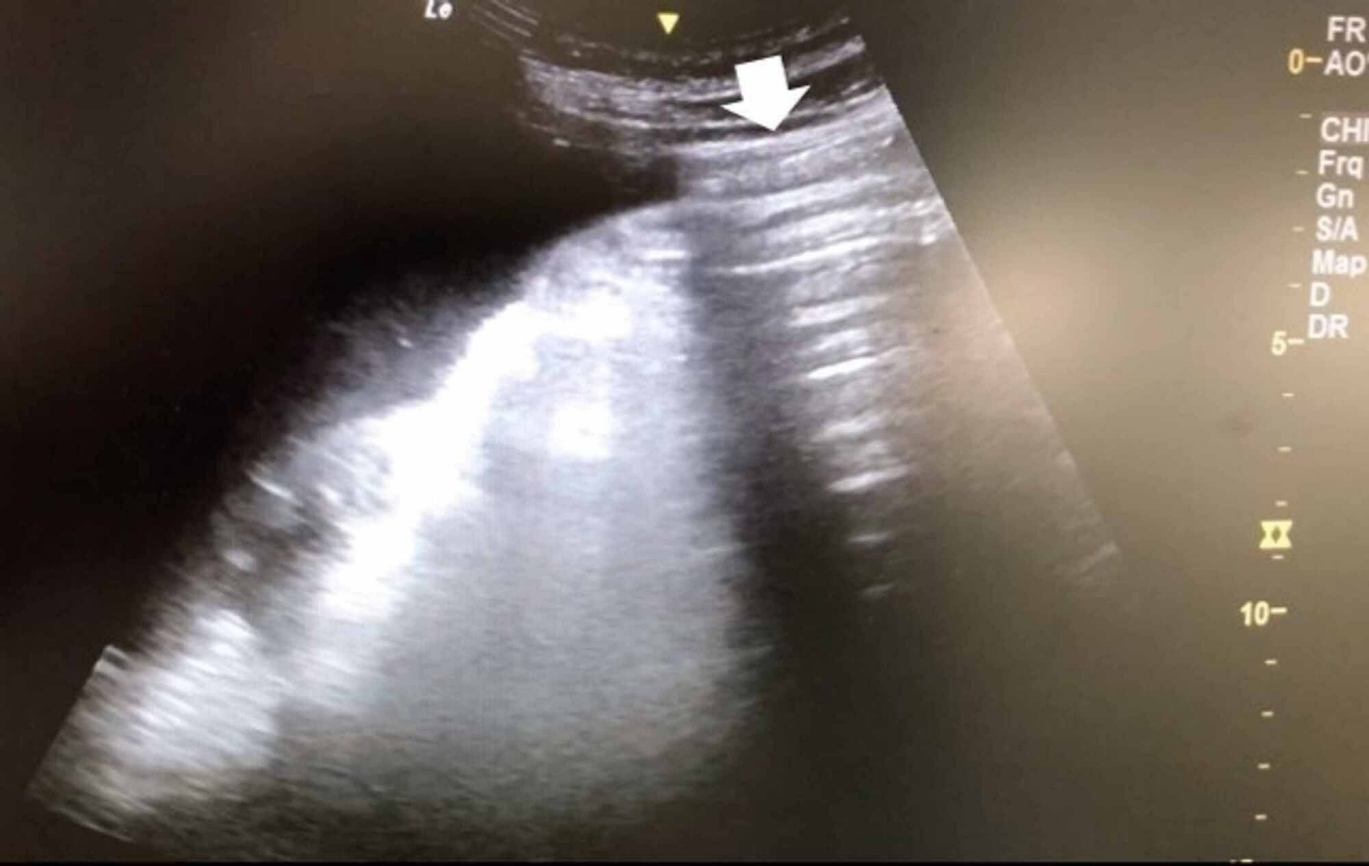
An enhanced peritoneal stripe sign

**Figure 3:**
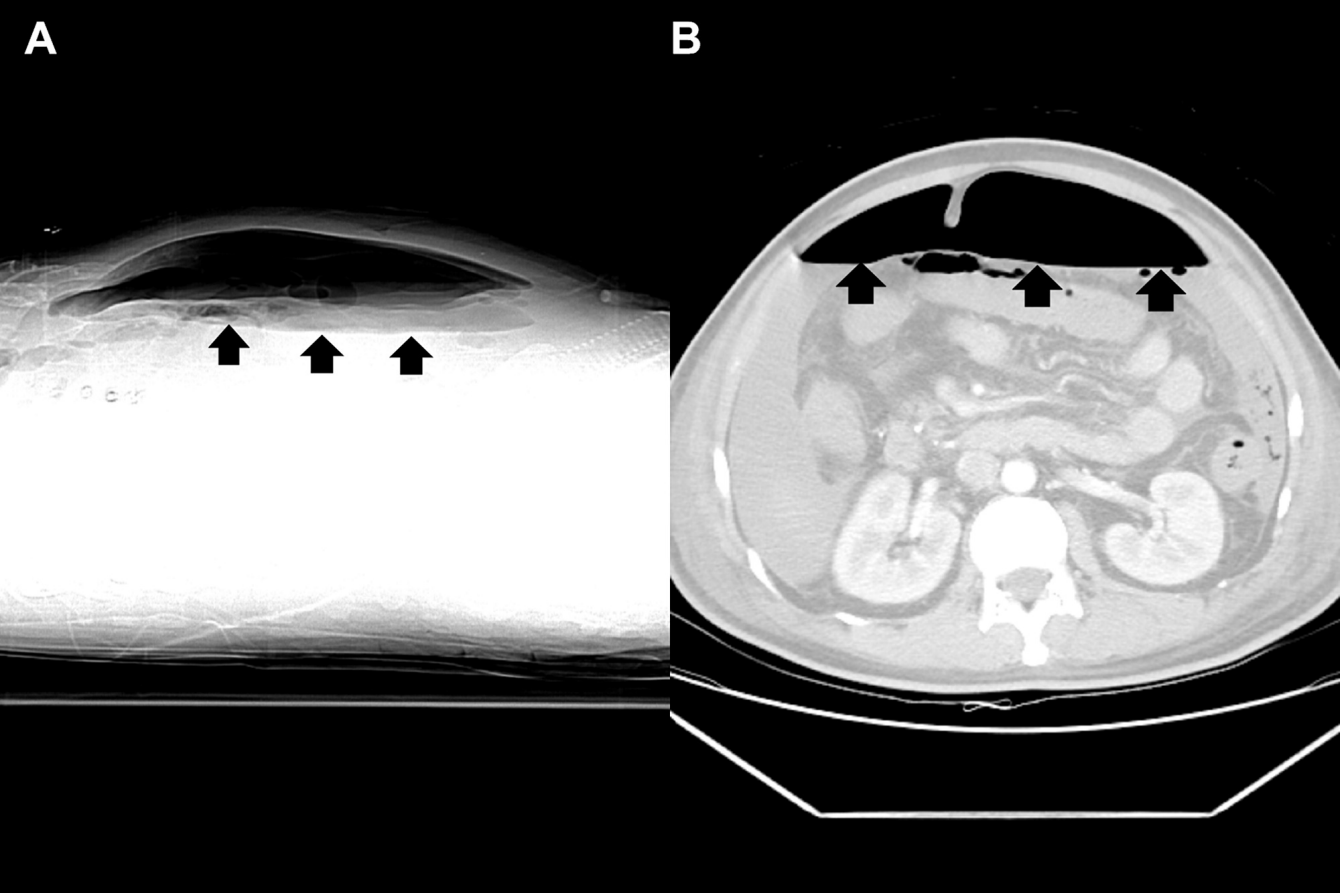
Intraperitoneal free air (black arrows) on abdominal computed tomography

Ten days after discharge, he started to have recurrent fever with worsening abdominal distension and hypotension. Physical examination upon admission revealed altered mental status and diffuse abdominal tenderness with muscle guarding. Laboratory data was notable for elevated white blood cell counts (WBC) of 52,200 /μL with 97.2% of neutrophils, alanine transaminase of 585 U/L, aspartate transaminase of 764 U/L, direct bilirubin of 2.4 mg/dL, and alkaline phosphatase of 600 IU/L. Abdominal x-ray revealed large oval radiolucency over the central part of the abdomen without interruption by intestine, a classical finding of pneumoperitoneum also known as “football sign” (Figure 1). An enhanced peritoneal stripe sign was noted on abdominal ultrasound (Figure 2, Video 1). Paracentesis yielded cloudy ascites with elevated WBC of 9077/μL with 96% of neutrophils, elevation of amylase of 585 U/L and lipase of 900 U/L. Abdominal CT demonstrated intraperitoneal free air (Figure 3), indicative of hollow organ perforation.

**Video 1:** Abdominal ultrasound: an enhanced peritoneal stripe sign

**Figure 4:**
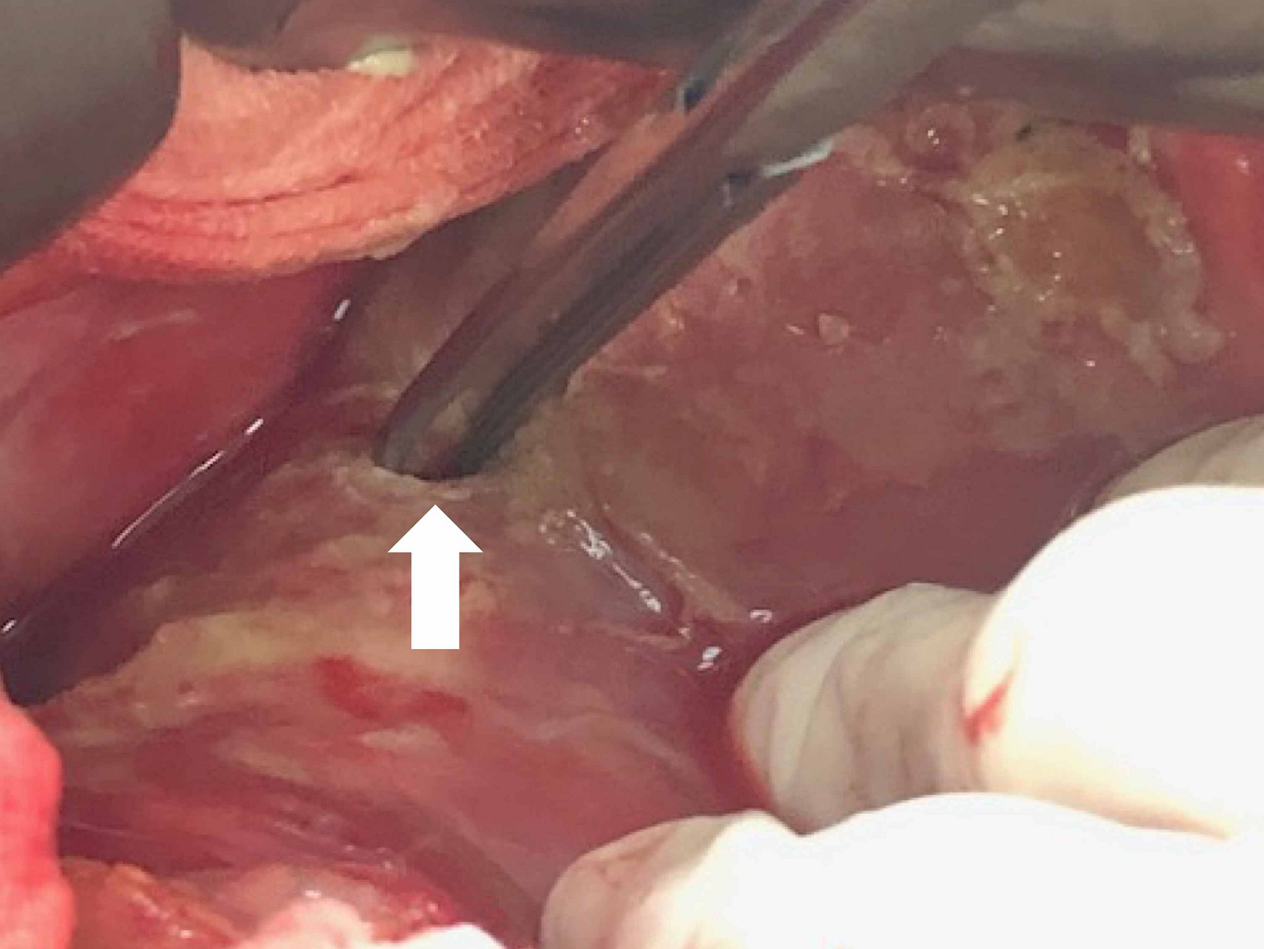
Perforation hole (white arrow) at anterior surface of gastric antrum.

Emergent laparotomy showed a 0.5 cm perforation at the anterior surface of the gastric antrum (Figure 4). Gastrorrhaphy, peritoneal irrigation, and feeding jejunostomy were performed and placed. Imipenem, micafungin, and daptomycin were administered empirically. Ascites culture later grew *Enterococcus faecium, Candida glabrata, *and *Candida albicans*. Despite timely management, the patient passed away on hospital day 23 due to progression of multiorgan failure.

## Discussion

Since the 1900s, plain film has become a valuable aid in diagnosing GI perforation. Although CT has been widely used to detect GI perforation due to its superior diagnostic accuracy [9], plain film is an inexpensive and fast modality that could offer valuable diagnostic information. Presence of pneumoperitoneum on upright or lateral decubitus abdominal radiograph is the typical finding of GI perforation [2]. Supine radiographs, while being less sensitive, may be the only acceptable radiographic projection for some patients with serious abdominal pain. Various free air signs on the supine radiographs were reported in previous literature, such as Rigler sign, hyperlucent liver sign, falciform ligament sign, urachus sign, and anterior superior oval sign [10]. Those signs result from free gas outlining the contour of different structures within peritoneal cavities.

Football sign was first described in 1960 by Miller et al. as one of radiographic patterns of perforated viscus in neonates [11]. It occurs when the free air accumulates in the anterior abdomen delineating the parietal peritoneum [3]. Occasionally, the longitudinal linear opacities could be observed from the outlining of the falciform and medial umbilical ligaments by free air. This specific radiographic pattern resembles an American football [7]. Multiple cases of football sign in neonates have been reported [4-7]. On the other hand, only one adolescent case [12] and one adult case [8] were noted in the literature. This is the first clear image reported of football sign in an adult. One possible reason is that a significant amount of air is required to accumulate within the peritoneal cavity so the contour of peritoneal cavity could be visualized. Neonates’ abdominal cavities are relatively small so less free air is needed to fill their abdominal cavities. Free air forms the radiolucent oval contour of a football, while the spine represents its laces. In our case, the gas bubble in the central part of the abdominal cavity can be easily observed due to the massive amount of underlying ascites.

## Conclusions

We presented a rare case of an adult patient who had gastric perforation and presented with a football sign on supine plain film. Recognition of the football sign can aid diagnosis and management of pneumoperitoneum in adult patients.
